# Sinusitis Orbitary Complications Classification: Simple and Practical Answers

**DOI:** 10.1016/S1808-8694(15)30116-6

**Published:** 2015-10-19

**Authors:** Richard Louis Voegels, Fabio de Rezende Pinna

**Affiliations:** Associate Professor of the University of São Paulo Medical School; Graduate student in Otorhinolaryngology at the University of São Paulo Medical School

Classifications for acute sinusitis orbitary complications have been characterized by incomplete theories and, often times, misaligned with anatomical principles. A correct classification for sinusitis complications is the foremost step towards a proper treatment of this set of clinical entities that bear a relatively high morbidity. In varied degrees, when not properly treated, sinusitis orbitary complications expose patients to amaurosis, cavernous sinus thrombosis and many other intracranial complications.

Despite a significant drop in complications we have seen throughout the last century - thanks to early antibiotic treatment, sinusitis complications have a constant prevalence in pediatric patients, and a varied and significant incidence among immunosuppressed patients. The growing increase in patient survival for those who suffer bone marrow, lung and liver transplants - thanks to intense secondary immunossupression, has made sinusitis complications an ever-present concern in the daily work of otorhinolaryngologists.

Since the first publication on this topic by Hubert in 1937, until current days, there are a number of misconceptions and much confusion insofar as clinical manifestations and complication extension correlations are concerned.

The first misconception was to state that cavernous sinus thrombosis would stem from orbitary complications. If the cavernous sinus is a structure primarily located in the cranial vault, why should it be grouped within the set of orbitary complications? Such question may even seem trivial at first, however this misconception remained undisputed since the famous publication by Chandler in 1970 and Maloney in 1987. It was only in 1997, with the work of Mortimore, that this complication was classified as an intracranial complication, and not orbitary.

Nonetheless, both Maloney in 1987, and Mortimore in 1997 insisted in using the terminology pre-septal and post-septal to classify orbitary complications.

By definition, the orbitary septum is but an eversion of the periorbitary bone tissue, representing an anatomical barrier, which separates the orbit from the upper and lower eyelids, making up the anterior border of the orbitary cavity. Thus, if the orbit, by definition, is behind the septum, wouldn’t it be a true incoherence to call it “pre-septal orbitary complication”? This led us to conclude that the expression pre-septal should be used for eyelid disorders, and it is not adequate to describe orbitary involvement. Thus, the terminology retroseptal and orbitary can be considered synonymous.

Once these concepts of pre-septal and post-septal are well understood, we can extend this discussion to another controversy created by Mortimore’s classification: Only the intrachoanal space is posterior to the orbitary septum? From this last publication of 1997, post-septal or orbitary complications are further broken down in intrachoanal and subperiosteal. The term subperiosteal abscess, as we see it, is very accurate, encompassing abscesses within the space between the papyraceous wall and the periorbital space. The term intrachoanal is related to the space surrounded by the orbit’s extrinsic muscles; extrachoanal is the space located between the cone and the periorbital space. In this classification there is no reference regarding the extrachoanal space, producing this misconception that within the retroseptal space there is only the intrachoanal space.

From the practical standpoint, in cases of subperiosteal abscess, besides this evidence of correlation between the papyraceous wall and the periorbital space, one can notice a smudging of the extrachoanal fat. Which would be the best classification for these cases? We believe that this situation is not present in any other classification.

Still regarding practical issues, often times it is difficult to tell an abscess from a phlegmon just by analyzing the CT scan. Nonetheless, such doubt should not be an obstacle for the decision of which is the best treatment approach when we face a case of sinusitis orbitary complication. Proper treatment stems from adequate clinical history, physical, endoscopic examination and a lot of experience in the interpretation of paranasal sinuses CT scans.

It is not easy to classify sinusitis orbitary complications and, unfortunately, there are still a number of incoherencies. In this issue, the paper called “Orbitary Complications of Acute Sinusitis: A New Classification” points out many issues pertaining to many classification systems that have been described in the literature since 1937. This study, besides describing clearly and precisely the anatomical principles, which are commonly presented in a confusing manner, also submits some practical and simple suggestions based on an elaborate concept about the real information that can be extracted from a CT scan. Could a classification based on three simple items such as orbitary cellulites, subperiosteal abscess and orbitary abscess be practical, objective, and yet free from incoherencies and uncertainties? It is certain that this paper will help to answer this and other questions mentioned in this editorial.

Disagreements and the constant search for more detailed explanations, supported by a constant updating on technological breakthroughs represent the cornerstone to establish a critical thinking for physicians inside or outside an academic institution. Thanks to this questioning and dynamic spirit, Brazilian Otorhinolaryngology has been evolving and increasingly gaining national and international appreciation.

